# Comparing predictors of sentence self-paced reading times: Syntactic complexity versus transitional probability metrics

**DOI:** 10.1371/journal.pone.0254546

**Published:** 2021-07-12

**Authors:** Bob Kapteijns, Florian Hintz

**Affiliations:** Max Planck Institute for Psycholinguistics, Nijmegen, the Netherlands; Tohoku University, JAPAN

## Abstract

When estimating the influence of sentence complexity on reading, researchers typically opt for one of two main approaches: Measuring syntactic complexity (SC) or transitional probability (TP). Comparisons of the predictive power of both approaches have yielded mixed results. To address this inconsistency, we conducted a self-paced reading experiment. Participants read sentences of varying syntactic complexity. From two alternatives, we selected the set of SC and TP measures, respectively, that provided the best fit to the self-paced reading data. We then compared the contributions of the SC and TP measures to self-paced reading times when entered into the same model. Our results showed that while both measures explained significant portions of variance in reading times (over and above control variables: word/sentence length, word frequency and word position) when included in independent models, their contributions changed drastically when SC and TP were entered into the same model. Specifically, we only observed significant effects of TP. We conclude that in our experiment the control variables explained the bulk of variance. When comparing the small effects of SC and TP, the effects of TP appear to be more robust.

## Introduction

The comprehension of written sentences consists of a multitude of low-level and high-level cognitive processes. During reading, the reader’s overall goal is to integrate incoming words into a coherent interpretation. The complexity of a sentence influences the speed with which it is read: Complex sentences are read more slowly than less complex sentences. An important topic in reading research has been the operationalization of sentential complexity. Previous research has led to two main approaches for quantifying complexity: in terms of syntactic complexity (SC), which refers to a set of measures based on hierarchical dependency structures (e.g., [1,2]), and in terms of transitional probability (TP), which refers to a class of information-theoretical metrics concerning probabilistic patterns of co-occurrence of linguistic units (e.g., [3,4]). Crucially, previous empirical reports have provided mixed evidence with regard to the importance of SC and TP in predicting sentence reading speed.

In the present study, we addressed this inconsistency and conducted a self-paced reading experiment featuring sentences of varying complexity. We first established for SC and TP separately the set of measures that best accounted for variability in participants’ sentence reading times. Then we compared the contributions of selected SC and TP measures to explaining variance in reading times, when entered into the same analysis. We discuss the implications for and the usefulness of SC and TP measures for quantifying reading behavior.

### Syntactic complexity

To investigate the effects of sentential complexity on reading behavior, a large body of psycholinguistic research has focused on specific, more complex or less complex syntactic constructions, including subject- and object-relative clauses, active and passive sentences, and syntactic ambiguities ([5–7], for reviews). The study of these constructions has been very popular, as they allow for tight experimental control. That is, more and less complex syntactic constructions (e.g. active and passive sentences) can often be formed using the same lexical materials, enabling researchers to compare processing costs associated with different syntactic constructions independent of lexical effects.

Complementary to studying specific sentence constructions, previous research has proposed measures for operationalizing syntactic complexity in a continuous fashion (e.g., [8–10]). Such measures of syntactic complexity (SC) capitalize on the fact that words that belong together (i.e., words that form interconnected syntactic dependencies) often do not appear in adjacent positions, but are distributed across the sentence. Such dependency structures (e.g., verb phrases, noun phrases, adjective phrases, etc.), consisting of non-adjacent lexical elements, are referred to as non-local hierarchical dependencies (e.g., [1,6,11]).

A common way of formalizing SC is the ‘left-branching’/‘right-branching’ (LB/RB) complexity metric (e.g., [9,12]). In LB structures, one or multiple dependents are encountered before its head, whereas in RB structures, the head is followed by its dependent(s) (see (1) for examples of left- and right-branching constructions).

(1) a. LB: My_dep3_ brother’s_dep2_ friend_dep1_ arrived_head_.
  b. RB: The dog slept_head_ on_dep1_ the doorstep_depd2_ of_dep3_ the house_dep4_ in which_dep5_ it_dep6_ lived_dep7_.

In both types of structures, open dependencies are created when the reader encounters a new, non-unified head or dependent. The process of integrating the encountered dependent(s) with their corresponding head (in case of LB), or a head with its dependent(s) (in case of RB), is crucial for understanding phrasal (sub-)structure. This process occurs once the last word of a dependency has been recognized and is often referred to as syntactic unification [2,13–16]. Syntactic unification cost, more commonly referred to as ‘syntactic complexity’ [17,18], increases when multiple open non-local dependencies need to be simultaneously kept active within working memory. A compelling body of behavioral studies has reported an association between high syntactic complexity and increased processing load, as reflected in longer self-paced reading or word fixation times (e.g., [17,19–24]). Moreover, such effects appear to be stronger for LB compared to RB dependency structures [8,21–23,25].

Tying in with a growing body of studies on the neurobiological mechanisms underlying syntactic processing (e.g., [26–30]), Uddén et al. [31] investigated functional brain activity associated with comprehending sentences varying in LB and RB complexity. They conducted a re-analysis of a functional magnetic resonance imaging dataset from Schoffelen et al. [32], where participants (n = 102) read stimulus sentences (n = 360) of varying syntactic complexity. Uddén et al. reported evidence for a left-hemispheric fronto-temporoparietal neural network involved in sentence comprehension that was particularly sensitive to variations in syntactic complexity. Their results also revealed that the neural effects for LB complexity were more pronounced than for RB complexity.

### Transitional probability

Fostered by the development of powerful computers and the availability of large linguistic corpora, there has been a rise in using information-theoretical metrics and computational modelling in linguistic research. Information-theoretic accounts of language processing often consider sentence comprehension a form of information processing, with individual words conveying specific amounts of information. The amount of information that is conveyed by a word is assumed to determine the cognitive load associated with comprehending it and with this word’s contribution to comprehending the entire sentence [3,33].

Transitional probability (TP) is a measure that defines word information in terms of probability characteristics that are based on statistical frequencies of sequential (co-)occurrence of words or phrases [3,33–36]. These probability measures can be derived from different types of probabilistic language models. For example, models may be trained on large amounts of input sequences whose syntactic structure may or may not be provided alongside the written word forms. As a result, probabilistic models differ as to whether or not they take the syntactic dependency information into account when calculating probability values for individual words.

TP measures are used to formalize the statistical probability of transitioning from one word to the next [3,36]. TP is commonly defined in terms of forward and backward TP (FTP and BTP): FTP refers to the probability that a particular word will follow a preceding context of one or more words. Hence, FTP captures how probable each word is given its previously encountered context. Conversely, BTP quantifies the probability that a certain context preceded the currently encountered word. Hence, BTP essentially refers to the probability of each word given its following word or string of words. To give an example, consider the sentence “I wish you a good weekend”: FTP can be used to quantify the probability that “weekend” will follow “(a) good”, while BTP is concerned with the probability that “good” has preceded the word “weekend”.

FTP and BTP are akin to the theoretical concepts of entropy and surprisal [3,35,37]. Less probable word transitions are typically associated with increased processing costs, resulting in higher (self-paced) reading times. Such effects have been observed frequently for FTP measures [33,36,38–40]. Studies investigating the effects of BTP are sparse and have reported mixed findings (e.g., Frank [40], found no effects on reading times; but see [41,42]).

### Comparing syntactic complexity and transitional probability metrics

Although studies of SC and TP are rooted in different theoretical assumptions and are operationalized using different methodologies, one goal of both approaches is to predict sentence comprehension difficulty. However, in spite of this common goal, previous research has often focused on one of the two approaches ([21–24]; see Hale [3] for review).

One attempt to assess and compare the predictive quality of SC and TP approaches in sentence comprehension was made by Frank and Bod [43]. Using fixation data from an eye-tracked reading experiment (Dundee corpus, [44]), the researchers investigated the degree to which TP estimates derived from three different types of language models explained word reading times. The three types of models were trained on materials taken from the Wall Street Journal corpus [45]. The first type of models were Markov models (also known as *n*-gram models); the second type of models were echo state networks (ESNs), a class of recurrent neural network (RNN) models. Both types of models relied solely on the sequential co-occurrence of words, and had no access to information about hierarchical syntactic dependencies in the text. The two types of models differed with regard to their maximal input length in that ESNs have no upper limit to the length of sentential context, whereas Markov models (by definition) do. The third type of models were probabilistic phrase-structure grammar (PSG) models. Unlike the other two model types, PSG models incorporated information about hierarchical syntactic structure when assigning probability values. The results obtained by Frank and Bod revealed that PSG models did not account for variance in reading times over and above the amount of variance explained by the sequential-structure models.

Using a similar approach in an electrophysiological study, Frank et al. [33] presented participants with sentences from the UCL corpus of reading times (see [40]). As before, the authors used three different types of language models to calculate their probability metrics: Markov (i.e., *n*-gram) models, RNN models, and probabilistic PSG models. As in Frank and Bod [43], only the latter type of models incorporated hierarchical syntactic dependency information. The results showed that reading individual words in the electrophysiological study elicited N400 components (event-related potential commonly associated with semantic processing [46]) that were strongly correlated with levels of surprisal (akin to FTP). Critically, the TP measures that were obtained from language models that did not include hierarchical structure (i.e., the Markov and RNN models) fitted the data better than the PSG models did. Based on these findings, Frank and colleagues concluded that hierarchical structure did not contribute significantly to explaining variance in the neural effects of sentence processing, complementing their earlier behavioral work (Frank & Bod, [43]).

In sum, in spite of the extensive body of literature showing effects of SC on reading (e.g., [17,20–24,47]), there is no consensus about the added value of incorporating information about hierarchical syntactic information into TP-based language models when predicting sentence comprehension difficulty. Note that in the studies by Frank and co-workers the measure resulting from each of the models that incorporated syntactic information (i.e., PSG models) were an *integration* of SC and TP. That is, a single value reflected the probability of a word taking into account syntactic structure *and* lexical co-occurrence frequency.

In the current study, we operationalized SC and TP as independent sets of measures and assessed and compared the predictive quality of SC and TP measures in self-paced sentence reading. This approach had the advantage that we could determine in independent analyses which SC and TP measures, respectively, provide the best fit to the data before pitting them against each other. Moreover, we could conduct correlation analyses between SC and TP measures to assess how much variance is shared between them—an analysis that is not possible when integrating SC and TP measures into one measure.

### The current study

We conducted a self-paced reading experiment and presented 73 participants with 160 sentences taken from the neuroimaging study by Schoffelen et al. [32] (see also Uddén et al. [31]; both did not record behavioral reading data) to obtain behavioral correlates of sentence reading (i.e., self-paced word reading times). We used the self-paced reading (SPR) paradigm as it has been used numerous times to study syntactic processing ([48,49], for reviews). Also, by presenting words in a serial fashion, we paralleled the setup used by Schoffelen et al. and Uddén et al. [31,32], who used rapid serial visual presentation in their fMRI study on the neural markers of SC as closely as possible.

We tested how well SC and TP measures predicted variance in participants’ self-paced reading times. Critically, instead of implementing hierarchical dependencies as part of a TP language model (as done by Frank and colleagues [33,43]; see also Fossum & Levy [47]), we operationalized SC and TP as two independent sets of measures, with the latter having no access to information about hierarchical syntactic structure. We opted for implementations of these measures that have previously shown effects on sentence reading performance. In particular, we calculated four SC measures: two left- and two right-branching ones (e.g., [19,20,23]), as well as four TP measures (FTP, BTP), calculated from an n-gram model trained on unanalyzed word sequences (e.g., [33,43,47]). In independent analyses, we first identified the sets of SC and TP measures, respectively, that provided the best fit to our self-paced reading data. Then we assessed the relative contributions of SC and TP measures to explaining variance in reading behavior by entering these sets into the same model. We conducted analyses both at the sentence- and the word-level. Although the main focus of the study was on comparing the effects of SC and TP, for sentence- and word-level analyses, we conducted models with and without control variables known to influence reading times (sentence/word length, word frequency, word position [50–53]). Note that most SPR studies focus on word-level analyses of reading times. Here, we complemented this approach with sentence-level analyses to capture the cumulative effects of SC and TP across the whole sentence.

The setup of the current study enabled us to replicate previous experiments investigating the effects of SC and TP on self-paced reading (e.g., [17,20–24,33,43,47]). Based on these reports, we predicted positive relationships between LB/RB complexity and reading times. Since we transformed our TP metrics to a positive scale, we also expected a positive relationship between FTP/BTP and reading times. Hence, we predicted longer reading times for more complex sentences (i.e., larger SC and TP values). The crucial question was whether SC would still explain a substantial portion of variance when entered simultaneously into an analysis with TP. If, as argued by Frank and colleagues [33,43], sentence comprehension difficulty is primarily explained by TP, this should not be the case. If, however, SC does contribute to explaining variance in sentence reading over and above TP, we should observe SC effects as main effects of the SC measures (in addition to main effects of TP).

## Method

### Participants

We tested 73 participants (60 female, mean age: 22.73). All participants were recruited from the participant pool of the Max Planck Institute for Psycholinguistics. Sixty participants were enrolled in (or had finished) university education, eleven were enrolled in higher vocational education (*HBO*) and two in intermediate vocational education (*MBO*). All participants were non-dyslexic native Dutch speakers and had normal or corrected-to-normal vision. All participants were naïve to the goal of the experiment. Written informed consent was obtained at the beginning of the session. As compensation for their participation, participants received 6 Euros. The ethics board of the Faculty of Social Sciences at Radboud University provided ethical approval to conduct the study.

### Materials

We selected 160 Dutch sentences from the stimuli used by Schoffelen et al. and Uddén et al. [31,32], that featured variable sentence length (ranging 9–15 words, average length: 11.46 words). The sentences were unconstrained in terms of syntactic structure and showed substantial variation in syntactic complexity. Note that we did not a priori control for the relationships between our measures of interest and/or the control variables. Instead, our focus was on obtaining a ‘natural’ spread in sentence length and complexity. Ninety-three sentences contained a relative clause. Capitals indicated the start of each sentence. The sentences did not contain punctuation or a full stop at the end.

### Syntactic complexity measures

Uddén et al. [31] formalized the LB and RB dependency structures based on dependency trees that were generated by an automated parser (FROG parser; [54]). These dependency trees were checked manually and adjusted if they contained errors. We used the per-word LB and RB values as calculated by Uddén et al., and calculated two additional syntactic complexity measures: the number of per-word left- and right-branching unifications (LB_unif and RB_unif). The dependency trees of two example sentences and an explanation of the calculation of the SC measures are provided in the supplementary materials S1 File.

#### LB and RB

The LB complexity value for each word was operationalized (see also Uddén et al. [31]) as the number of left-branching dependencies that were (1) opened, (2) unified (i.e., closed) or (3) remained open at that particular point in the sentence. That is, as the sentence unfolded from left to right, a word’s LB value was equivalent to the number of dependents that had been encountered and that could not yet be attached to a verbal head. The LB measure thus incorporated all syntactic dependencies of a given word in a sentence and the processing costs associated with them. Analogously to the LB measure, each word’s RB complexity value was operationalized as the number of right-branching dependencies that were opened, unified or remained open at the occurrence of that word in the sentence.

#### LB_unif and RB_unif

Both unif measures were subsets of their respective LB and RB counterparts. The LB_unif measure reflected the number of left-branching unifications that occurred at each word (if any) in the sentence. Thus, this measure differed from the LB measure in that it only considered dependencies that were *unified* at a given word, and neglected the dependencies that were opened or remained open. Analogously, a word’s RB_unif value reflected the number of right-branching dependencies that were unified at that word in the sentence. The inclusion of both unif measures was motivated by previous reports that showed substantial processing costs associated specifically with the operation of unifying a syntactic head with its dependent(s) (see [19,24,30]).

All four SC measures were defined for all words in each sentence (including e.g. auxiliaries and determiners). Words could receive a value of zero in case no dependencies or unifications were present. As described above, we performed sentence-level and word-level analyses. The dependent variable in the sentence-level analysis was obtained by summing the reading times of all words in a given sentence. We operationalized the LB, RB, LB_unif and RB_unif complexity values for each sentence as the sum of the values of all words in that sentence. Figs 2 and 3 show the (Pearson) correlation heatmaps for all predictors at the sentence- and word-level, respectively. As can be seen, LB and LB_unif as well as RB and RB_unif were quite highly correlated, which is to be expected given that one is a subset of the other. Correlations between left- and right-branching measures (also for the unif measures) were negative, indicating that high left-branching complexity often coincided with low right-branching complexity and vice versa. Note also that word- and sentence-level correlations were quite different. For example, at the word-level, the positive correlations between LB/RB and their respective unif measures were less strong. Moreover, while the correlation between LB and RB changed slightly from the sentence- (r = 0.09) to the word-level (r = 0.23), it flipped for the correlation between LB_unif and RB_unif (sentence: r = -0.44, word: r = 0.31).

### Transitional probability measures

Our TP measures included bigram and trigram forward and backward TP, obtained from an n-gram model that was trained on unanalyzed word sequences and did not incorporate information about hierarchical sentential syntax ([55]). In line with previous studies (e.g., [4,33,36]), the four TP measures were operationalized as the logarithm of each word’s occurrence probability.

#### Forward and backward bigram TP

Bigram TP refers to the probability of transitioning from one word to another. Forward bigram TP (bigram FTP), more specifically, refers to the probability of encountering the current word given its preceding (one-word) context. Backward bigram TP (bigram BTP), on the other hand, refers to the probability with which a certain one-word context has preceded the current word. Bigram TP could not be calculated for the first word in each sentence.

#### Forward and backward trigram TP

To capture slightly longer stretches of text, we included trigram TPs, where forward trigram TP (trigram FTP) refers to the probability of the current word given the preceding two-word context and backward trigram TP (trigram BTP) refers to the probability that a certain two-word context has preceded the current word. Trigram TP was not calculated for the first two words in each sentence.

For the sentence-level analyses, the four TP measures were summed for all words in a given sentence. All TP measures were provided on a positive scale, with larger values reflecting more improbable (i.e., unexpected/surprising) word transitions.

As shown in Figs 2 and 3, forward bigram and trigram TP were moderately to strongly correlated, both at the sentence- and word-level, as were backward bigram and trigram TP. This is to be expected given that bigrams are included in trigrams. Furthermore, the two bigram and the two trigram measures were strongly correlated at the sentence-level (due to summation), but not at the word-level.

### Control variables

In addition to the four SC and four TP measures, we included multiple control variables in our sentence-level and word-level analyses. For the sentence-level analyses, we included the number of words (NWords) and summed word frequency of all words in a given sentence (SumFreq; retrieved from SUBTLEX-NL [56], and converted to the Zipf scale [57]). At the word-level, we included word length (operationalized as number of letters; NLetters (e.g., [58]), word frequency (Zipf) and word position (running word number within the sentence).

Table 1 shows the descriptive statistics of all predictors at the sentence-level, summed across all words per sentence. Table 2 provides the same overview for the word-level predictors (except word position).

**Table 1:** Descriptive statistics of sentence-level predictors (n = 160; all measures summed per sentence).

Measure	Mean	SD	Range
NWords	11.46	1.32	9–15
SumFreq	61.79	8.35	45.41–86.93
LB	20.51	7.24	8–41
RB	14.91	4.71	7–30
LB_unif	6.54	1.39	4–11
RB_unif	3.86	1.30	2–7
Forward bigram TP	30.93	4.53	19.21–43.85
Forward trigram TP	17.92	3.97	9.11–27.99
Backward bigram TP	29.27	4.65	16.59–40.23
Backward trigram TP	44.75	7.10	30.38–63.95

**Table 2:** Descriptive statistics of word-level predictors.

Measure	Mean	SD	Range
NLetters	4.96	2.51	1–13
Zipf	5.39	1.62	1.30–7.60
LB	1.79	1.31	0–6
RB	1.30	0.72	0–4
LB_unif	0.57	0.82	0–4
RB_unif	0.34	0.48	0–1
Forward bigram TP	2.97	1.41	0–7.67
Forward trigram TP	1.90	1.44	0–6.56
Backward bigram TP	2.81	1.63	0.03–7.68
Backward trigram TP	4.74	1.75	0.39–7.68

### Procedure

The experiment was carried out at the Max Planck Institute for Psycholinguistics. Participants were tested individually, seated in an experiment booth, in front of a computer screen. They were instructed to read the sentences silently as fast as possible while still being able to comprehend their contents. Each sentence was presented word by word, using a non-cumulative, stationary window self-paced reading paradigm. Each word appeared in the center of the screen. The participants pressed the space bar to bring up the next word, which replaced the previous word. Reading times (RTs; the difference between onset of word presentation and the button press) were recorded for each word in every sentence.

To ensure that participants kept focus while reading the sentences, 20% (32 out of 160) of the sentences were followed by a yes/no question. The questions focused on the wording of the sentence (e.g., “was the word X mentioned?”), or the semantic content (e.g., “was person A angry with person B?”). The correct answer was ‘yes’ for half of the questions.

All participants read all 160 sentences. The order of sentences was random and different for each participant. After reading a sentence, participants pressed the Enter key to start the next sentence. The entire task consisted of four blocks (each containing 40 trials), which were divided by small breaks. In total, the experiment took approximately 25 minutes.

### Data pre-processing

Prior to statistical analysis, we excluded two participants whose accuracy on the yes/no questions was below 80% (same criterion as [33,58]). Subsequently, the RT data were screened for outliers. In line with previous literature [58,59], all sentence trials that contained word RTs shorter than 100 ms or longer than 2,000 ms were excluded. This led to the exclusion of 2.68% of all trials. The RTs of all words were log-transformed. For the sentence-level analyses, all word RTs were summed (and then log-transformed) to obtain one RT per sentence per participant.

**Fig 1:**
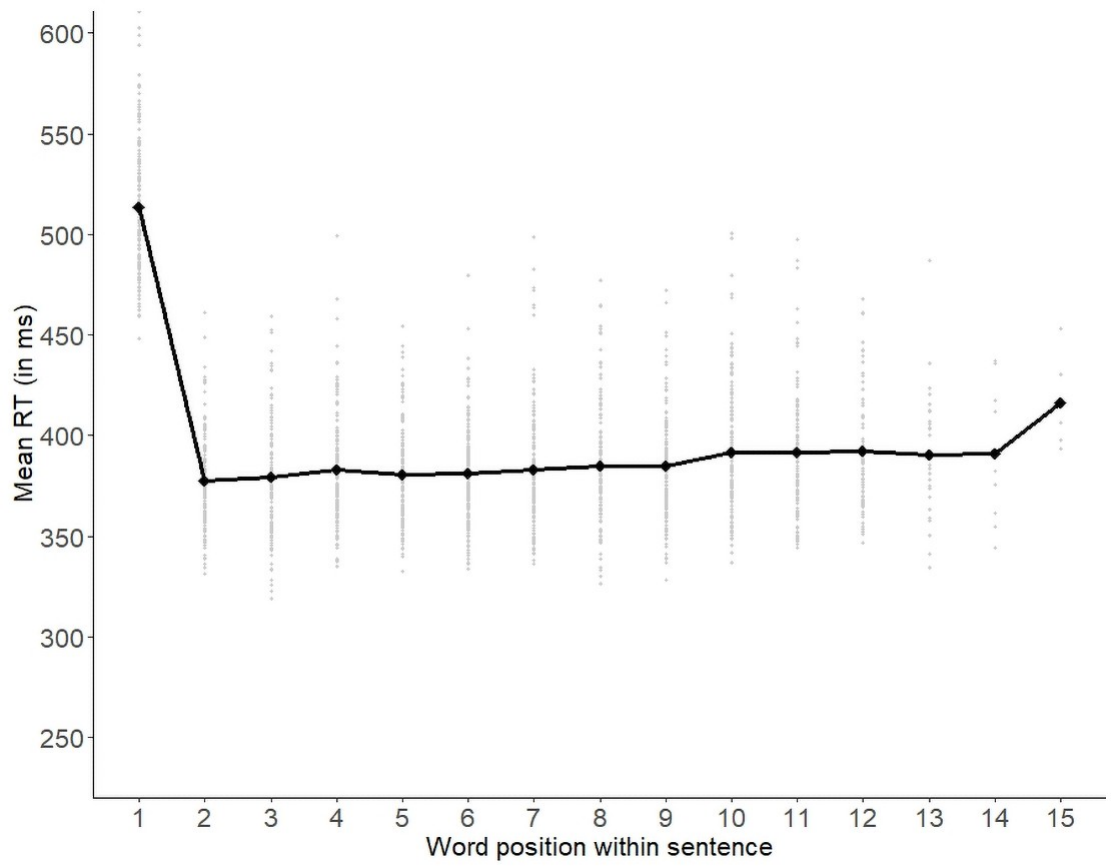
Average word RTs by word position. Black dots represent average RTs for each word position. Gray dots represent average RTs per word per sentence. Note that only five sentences had a length of fifteen words.

We plotted the average RT by word position over all participants (Fig 1). This plot revealed that the first word in each sentence was read substantially more slowly (i.e., on average by more than 100 ms) than the following words. As the SC and TP measures for the first word in a sentence are naturally very low or even undefined, such outlier RTs could confound our analyses. We therefore excluded the first word of each sentence from all subsequent analyses. This did not affect any of the TP measures, as the sentence-initial words had not been included in the measures (see ‘Transitional probability measures’ section). With regard to the SC measures and word frequency, there were some minimal changes to the sentence-level means (LB: *M* = 19.52, RB: *M* = 14.68, no change for LB_unif and RB_unif, SumFreq: M = 55.39). Similarly, the word-level means changed slightly as compared to the means reported in Table 2 (LB: *M* = 1.87, RB: *M* = 1.40, LB_unif: *M* = 0.63, RB_unif: *M* = 0.37, Word Zipf: *M* = 5.30).

## Results

**Fig 2:**
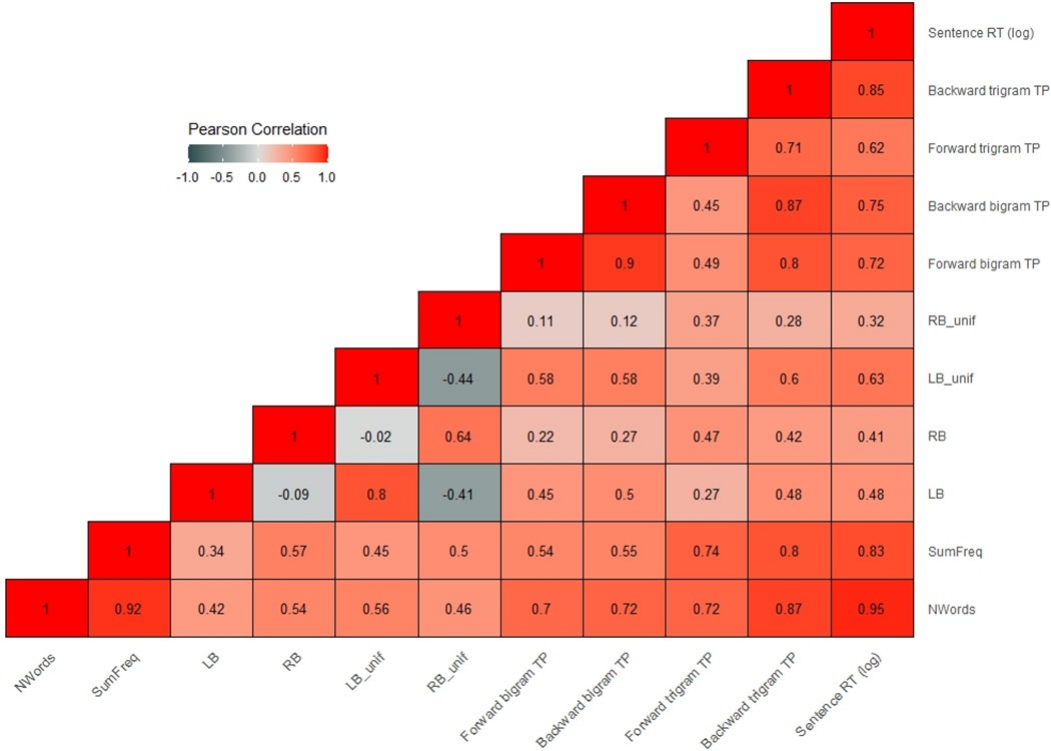
Heatmap showing the Pearson correlations between all sentence-level predictors and sentence RTs.

**Fig 3:**
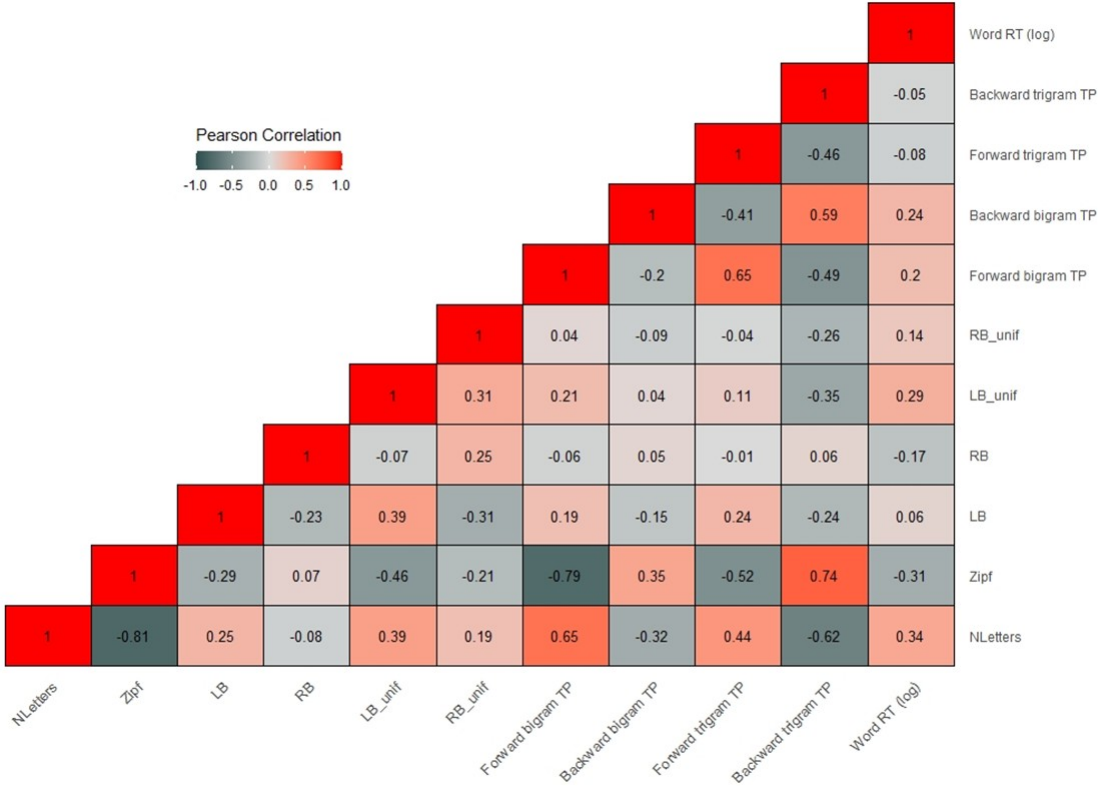
Heatmap showing the Pearson correlations between all word-level predictors and word RTs. Note: The TP measures contained some missing values, as by definition, the first word of a sentence is not defined in bigrams and the first two words are not defined in trigrams. Hence, bigram and trigram measures did not contain values for the first (and second) word(s) of each sentence.

The average response accuracy to the yes/no comprehension questions (after exclusion of two participants) was 93.1%. After outlier removal, the average reading time per sentence (over all participants) was 4529 ms (SD = 1621, range = 1747–15490 ms). Across all sentences and all participants, the average per-word reading time was 385 ms (SD = 170, range 100–1984). The heatmaps in Figs 2 and 3 contain the correlations between sentence and word RTs and the various predictors.

The heatmaps show that the strongest correlations were observed between sentence/word RTs and Nwords/NLetters and SumFreq/Zipf (i.e., the control variables). Note that at the sentence-level SumFreq and sentence RT correlated positively, whereas a negative correlation would be expected (frequent words leading to shorter RTs). The positive correlation is most likely an artifact of the summation of Zipf values.

At the sentence-level, all of our measures of interest showed moderate to strong positive correlations with sentence RTs. At the word-level, LB_unif, forward and backward bigram TP showed the strongest positive correlation ranging between r = 0.2 and r = 0.29.

### Control measures

Prior to assessing the contribution of the predictors of interest, we assessed the contribution of the control variables to explaining variance in RTs. To that end, we fitted two linear-mixed effects models: one sentence-level and one word-level model in R (R Development Core Team, 2011), using the lme4 package [60]. The sentence-level model contained ‘participant’ and ‘sentence’ as random effects; at the word-level, these were ‘participant’ and ‘word’ (all random effects had random intercepts). The dependent variable was log-transformed sentence/word RTs.

At the sentence-level, the model additionally contained NWords and SumFreq as continuous predictors; at the word-level the model contained NLetters, Zipf and word position. All continuous predictors were scaled and centered. Given the sample size of our dataset and the number of items each participant read, we consider t-values larger than +/- 2 to be statistically significant [61].

As shown in Table 3, at the sentence-level we observed significant contributions of both NWords and SumFreq. That is, longer sentences and sentences composed of less frequent words resulted in longer RTs than sentences containing fewer and more-frequent words. At the word-level, NLetters and word position showed significant positive effects, such that word RTs were longer for longer than for shorter words and such that words later in the sentence (larger word position value) were read more slowly than words earlier in the sentence. Zipf frequency did not contribute significantly to word RTs.

**Table 3:** Results of the mixed-effects model with only control predictors.

	Sentence-level			Word-level		
Predictor	Estimate	SE	t	Estimate	SE	t
(Intercept)	3.634	0.013	280.81	2.554	0.013	191.77
NWords/NLetters	0.064	0.003	19.91	0.006	0.001	6.25
SumFreq/Zipf	-0.018	0.003	-5.51	-0.001	0.001	-1.33
word position	-	-	-	0.008	0.001	13.29

Sentence-level: Number of obs.: 11055, groups: Sentence, 160; Participant, 71.

Word-level: Number of obs.: 115583, groups: Word, 1673; Participant, 71.

### Syntactic complexity

To estimate the variance explained by SC measures (LB/RB vs. LB_unif/RB_unif) in addition to that explained by the control variables and to determine which set of SC measures provided the best fit to the data, we fitted four linear mixed-effects models (two word- and two sentence-level models), which were identical in structure to the previous models, but additionally contained one of the two sets of SC variables (either LB and RB or LB_unif and RB_unif, scaled and centered).

Table 4 summarizes the results of the four SC models. As in the previous sentence-level model, we observed significant effects of NWords and SumFreq, with longer sentences and sentences composed of less frequent words resulting in longer RTs than shorter sentences and sentences containing frequent words. With regards to the SC measures, we found that LB showed a marginal effect, with sentences containing more complex left-branching structures being read more slowly than sentences with less complex left-branching structures. RB showed a negative effect suggesting that sentences with larger RB values were read faster. Neither LB_unif nor RB_unif showed a significant effect at the sentence-level.

**Table 4:** Results of the mixed-effects models concerning syntactic complexity (SC).

		Sentence-level				Word-level			
Model	Predictor	Estimate	SE	t	VIF	Estimate	SE	t	VIF
*LB & RB complexity*	(Intercept)	3.633	0.013	280.92		2.55	0.013	191.77	
	NWords/NLetters	0.062	0.003	19.20	8.14	0.006	0.001	6.08	2.92
	SumFreq/Zipf	-0.014	0.003	-4.31	8.22	-0.001	0.001	-1.36	2.97
	word position	-	-	-		0.008	0.001	13.12	1.06
	LB	0.003	0.001	1.92	1.48	-0.001	0.001	-0.71	1.19
	RB	-0.004	0.002	-2.65	1.87	-0.002	0.001	-4.06	1.06
*LB_unif & RB_unif*	(Intercept)	3.634	0.013	280.91		2.55	0.013	191.77	
	NWords/NLetters	0.064	0.005	12.37	20.79	0.006	0.001	6.15	2.91
	SumFreq/Zipf	-0.014	0.003	-4.38	8.09	<0.001	0.001	0.14	3.11
	word position	-	-	-		0.007	0.001	11.76	1.19
	LB_unif	-0.001	0.004	-0.11	13.25	0.003	0.001	5.40	1.33
	RB_unif	-0.006	0.004	-1.54	11.46	<0.001	0.001	0.10	1.31

Model 1: Sentence-level: LB & RB. Number of obs: 11055, groups: Sentence, 160; Participant, 71.

Model 2: Sentence-level: LB_unif & RB_unif. Number of obs: 11055, groups: Sentence, 160; Participant, 71.

Model 3: Word-level: LB & RB. Number of obs: 115583, groups: Word, 1673; Participant, 71.

Model 4: Word-level: LB_unif & RB_unif. Number of obs: 115583, groups: Word, 1673; Participant, 71.

In the word-level analyses, the control variables NLetters and word position showed significant positive effects (i.e., longer RTs for longer words and words later in the sentence). While LB did not contribute significantly to explaining variance in word RTs, RB showed a negative effect with words with larger right-branching values (right-branching dependencies being opened, kept open or closed) being read faster than words with fewer right-branching dependencies. In contrast, the model that contained the two unif measures revealed a positive effect of LB_unif such that words where more left-branching dependencies were closed (i.e., unified) were read more slowly than words where fewer left-branching dependencies were closed. RB_unif showed no effect.

It should be highlighted that while the SC predictors showed some significant effects, the bulk of variance in both sentence- and word-level RTs was explained by the control variables (i.e., sentence/word length, frequency and word position), as reflected in the estimates in Table 4. Given that the SC measures were moderately correlated with the control variables (see heatmaps in Figs 2 and 3), multicollinearity could have been an issue. Including multiple correlated predictors in the same model may result in biased coefficients [62]. In fact, in some cases, multicollinearity may even reverse the directionality of effects: Recall that—based on previous research—we predicted positive effects (larger SC values associated with longer RTs), but that at the sentence- and word-level, RB had negative effects in the models described above.

To assess to what extent multicollinearity was an issue in our four models, we calculated variance inflation factor (VIF) values of our predictors (see Table 4). VIF values reflect the degree to which the variance explained by one predictor is inflated due to multicollinearity effects. Generally, predictors with VIF values that exceed 5 are regarded as problematic in linear models [63,64], and it is advised to remove them as the information they code is redundantly contained. We found high VIF values at the sentence-level for NWords and for SumFreq.

To assess the contributions of the SC predictors (our measures of interest) to RTs, independent of the control variables, we re-ran the models described above. To facilitate the comparison between sentence- and word-level models, we re-fitted all four models, removing the control variables. The results are shown in Table 5. At the sentence-level, both sets of SC measures showed significant positive effects: larger LB/RB/LB_unif/RB_unif values were associated with longer RTs. The estimates of the unif measures were larger than those of the corresponding LB/RB measures. At the word-level, both unif measures had significant positive effects. The effects of LB and RB were both negative; the effect of LB was not significant.

**Table 5:** Results of the mixed-effects model with only SC predictors.

	Sentence-level				Word-level			
Predictor	Estimate	SE	t	VIF	Estimate	SE	t	VIF
(Intercept)	3.634	0.013	274.58		2.554	0.013	191.73	
LB	0.027	0.003	8.74	1.01	-0.001	0.001	-0.55	1.05
RB	0.023	0.003	7.71	1.01	-0.003	0.001	-4.12	1.05
(Intercept)	3.634	0.013	279.77		2.554	0.013	191.75	
LB_unif	0.048	0.002	26.71	1.23	0.006	0.001	8.54	1.10
RB_unif	0.037	0.002	20.67	1.23	0.003	0.001	5.35	1.10

Sentence-level: LB/RB: Obs.: 11055, groups: Sentence, 160; Participant, 71.

Sentence-level: unifs: Obs.: 11055, groups: Sentence, 160; Participant, 71.

Word-level: LB/RB: Obs.: 115583, groups: Word, 1673; Participant, 71.

Word-level: unifs: Obs.: 115583, groups: Word, 1673; Participant, 71.

The results of the SC-only models show that multicollinearity influenced (some of) the effects of the SC predictors. Given the fact that the unif measures showed more consistent effects throughout the various models (with and without control variables) and had larger estimates, we selected LB_unif and RB_unif for our ‘full-model’ analysis, where we compared the predictive power of SC and TP predictors.

### Transitional probability

To estimate the contribution of the TP measures to sentence and word RTs and to determine which set of TP measures (bigram or trigram) provided the best fit to the data, we adopted a similar approach as for the SC measures. As a first step, we fitted four models, two sentence- and two word-level models, which contained control and TP predictors. Table 6 summarizes the results. In all four models, we observed large positive effects of length (NWords and NLetters, respectively), a negative effect of frequency and—at the word-level—a positive effect of word position. Regarding our measures of interest, we observed a significant positive effect of bigram and trigram BTP (i.e., longer reading times for more unexpected backward-looking transitions), both at the sentence-level and the word-level. In both sentence-level models, forward TP showed trends for a negative effect; in the word-level models, these negative effects were statistically significant suggesting that words with larger forward bigram/trigram TP were read faster than words with lower forward TP.

**Table 6:** Results of the mixed-effects models concerning transitional probability (TP).

		Sentence-level				Word-level			
Model	Predictor	Estimate	SE	t	VIF	Estimate	SE	t	VIF
*Bigram BTP & FTP*	(Intercept)	3.634	0.013	280.86		2.55	0.013	191.89	
	NWords/NLetters	0.061	0.004	14.64	12.97	0.007	0.001	8.22	2.95
	SumFreq/Zipf	-0.017	0.004	-4.82	9.38	-0.008	0.001	-7.20	4.83
	word position	-	-	-	-	0.007	0.001	13.78	1.01
	Bigram FTP	-0.004	0.003	-1.44	6.02	-0.005	0.001	-5.29	2.86
	Bigram BTP	0.007	0.003	2.71	5.62	0.011	0.001	20.18	1.18
*Trigram BTP & FTP*	(Intercept)	3.634	0.013	280.88		2.56	0.013	193.69	
	NWords/NLetters	0.058	0.004	15.41	10.66	0.007	0.001	7.23	2.98
	SumFreq/Zipf	-0.015	0.003	-4.57	8.58	-0.012	0.001	-10.17	4.28
	word position	-	-	-	-	0.006	0.001	9.99	1.02
	Trigram FTP	-0.004	0.002	-1.97	2.57	-0.007	0.001	-10.97	1.41
	Trigram BTP	0.008	0.002	3.22	4.48	0.010	0.001	12.18	2.26

Model 1: Sentence-level: Bigram. Number of obs: 11055, groups: Sentence, 160; Participant, 71.

Model 2: Sentence-level: Trigram. Number of obs: 11055, groups: Sentence, 160; Participant, 71.

Model 3: Word-level: Bigram. Number of obs: 115167, groups: Word, 1667; Participant, 71.

Model 4: Word-level: Trigram. Number of obs: 104248, groups: Word, 1509; Participant, 71.

As for the SC models, we calculated VIF values for the predictors in our four TP models. We found that in both sentence-level models the control variables had VIF values far above 5. Moreover, in the bigram sentence-level model, forward and backward TP predictors also had values above 5. None of the predictors in the word-level models were affected by multicollinearity.

As for the SC analyses, we re-ran the four TP models to estimate the contributions of TP predictors independent of the control variables (Table 7). As in the SC-only models, removing the control variables drastically changed the effects of the TP predictors. Forward and backward bigram TPs showed significant positive effects, both at the sentence- and the word-level. While backward trigram TP had a significant positive effect on sentence RTs, there was no hint of an effect of forward trigram TP. Both trigram measures had significant negative effects in the word-level analysis. Thus, given the more consistent effects of bigram TP, we selected these measures for the full-model analysis that compared the contributions of SC and TP directly.

**Table 7:** Results of the mixed-effects model with only TP predictors.

	Sentence-level				Word-level			
Predictor	Estimate	SE	t	VIF	Estimate	SE	t	VIF
(Intercept)	3.634	0.013	276.21		2.554	0.013	191.84	
Bigram FTP	0.013	0.006	2.11	5.07	0.006	0.001	9.93	1.05
Bigram BTP	0.026	0.006	4.41	5.07	0.009	0.001	13.85	1.05
(Intercept)	3.634	0.013	278.37		2.555	0.013	193.69	
Trigram FTP	0.002	0.003	0.76	2.04	-0.004	0.001	-5.18	1.27
Trigram BTP	0.042	0.003	13.98	2.04	-0.002	0.001	-2.02	1.27

Sentence-level: Bigram: Obs.: 11055, groups: Sentence, 160; Participant, 71.

Sentence-level: Trigram: Obs.: 11055, groups: Sentence, 160; Participant, 71.

Word-level: Bigram: Obs.: 115167, groups: Word, 1667; Participant, 71.

Word-level: Trigram: Obs.: 104248, groups: Word, 1509; Participant, 71.

### Full-model: SC versus TP

To assess the relative contributions of SC and TP measures to explaining variance in self-paced reading times, we fitted one sentence-level and one word-level model, containing the (summed) LB_unif, RB_unif, bigram FTP and bigram BTP measures. The full sentence-level model contained NWords and SumFreq, and the full word-level model contained word length, Zipf and word position as control predictors (all scaled and centered). Both models had ‘participant’ and ‘sentence’/‘word’ (both with random intercepts) as random effects.

Table 8 summarizes the results of the two models. As in the previous models, we observed that sentence/word length and frequency had effects in the expected directions. Also, as before, word position had a positive effect at the word-level, such that words later in the sentence were read more slowly than words early in the sentence. With regards to our measures of interest, at the sentence-level, there was a significant positive effect of bigram BTP and a trend for a negative effect of bigram FTP. Neither LB_unif nor RB_unif had a significant effect. At the word-level, bigram BTP showed a significant positive effect, whereas bigram FTP showed a significant negative effect. The two unif measures did not show a substantial contribution to explaining word RTs.

**Table 8:** Results of the ‘full’ model at the sentence-level and word-level.

	Sentence-level				Word-level			
Predictor	Estimate	SE	t	VIF	Estimate	SE	t	VIF
(Intercept)	3.634	0.013	280.93		2.554	0.013	191.89	
NWords	0.063	0.006	10.72	27.20	0.007	0.001	8.19	2.96
SumFreq	-0.015	0.004	-4.29	9.70	-0.008	0.001	-6.39	5.99
word position	-	-	-	-	0.007	0.001	12.66	1.19
LB_unif	<0.001	0.004	0.04	13.33	0.001	0.001	0.18	1.53
RB_unif	-0.005	0.004	-1.26	11.78	< -0.001	0.001	-0.06	1.34
Bigram FTP	-0.004	0.003	-1.63	6.04	-0.005	0.001	-4.90	3.27
Bigram BTP	0.006	0.003	2.21	5.78	0.011	0.001	19.34	1.27

Sentence-level: Obs: 11055, groups: Sentence, 160; Participant, 71.

Word-level: Obs: 115167, groups: Word, 1667; Participant, 71.

As in the previous analyses, the sentence-level control predictors had VIF values larger than five. To complement the previous analyses and to compare the contributions of SC and TP measures independent of any influence from the control predictors, we re-ran the ‘full’ model containing only the variables of interest. The results of this model are listed in Table 9. Removing the control variables had again dramatic effects on the contributions of SC and TP measures: With one exception (bigram FTP at the sentence-level), all SC and TP predictors showed significant positive effects (higher complexity/more improbable word combinations associated with longer RTs) in both sentence-and word-level analyses.

**Table 9:** Results of the ‘full’ model, without control predictors.

	Sentence-level				Word-level			
Predictor	Estimate	SE	t	VIF	Estimate	SE	t	VIF
(Intercept)	3.634	0.013	280.07		2.554	0.013	191.86	
LB_unif	0.039	0.002	16.35	2.56	0.004	0.001	6.45	1.17
RB_unif	0.032	0.002	16.45	1.68	0.004	0.001	6.83	1.11
Bigram FTP	0.001	0.003	0.23	5.29	0.005	0.001	8.52	1.11
Bigram BTP	0.010	0.004	2.92	5.44	0.008	0.001	14.07	1.06

Sentence-level: Obs: 11055, groups: Sentence, 160; Participant, 71.

Word-level: Obs: 104248, groups: Word, 1667; Participant, 71.

## Discussion

The main goal of the present study was to assess the relative contributions of SC and TP to explaining variance in reading times. We conducted a self-paced reading experiment where native Dutch participants read sentences of varying complexity. We conducted mixed-effects model analyses at the sentence- and word-level and identified, in independent analyses, which set of SC and TP measures, respectively, provided the best fit to the data.

These analyses revealed significant contributions of the SC measures to explaining variance in RTs. Our results thus replicate earlier research showing that SC, operationalized in a continuous fashion, predicts sentence reading difficulty (e.g., [17,20–24,47]). Moreover, these results complement the neurobiological work by Uddén et al. [31], who reported evidence for a left-hemispheric fronto-temporoparietal neural network involved in sentence comprehension that was sensitive to variations in syntactic complexity. Apart from answering occasional comprehension questions, the participants in Uddén et al.’s study did not carry out a behavioral task. Since we used a subset of their materials and a similar design (rapid serial visual presentation in the fMRI study and non-cumulative stationary window self-paced reading in the present study), the present results fill that gap and demonstrate an association between SC and behavioral processing costs in reading. Note, however, that Uddén et al.’s analyses were based on the LB and RB and not the unif measures. As explained in the Introduction, another goal of the present study was to compare LB/RB with the unif measures. Our analyses revealed that the unif measures provided a better and more consistent fit to the data (across multiple analyses) than the LB/RB measures. In other words, SC measures indexing the number of syntactic unifications occurring at a given word were better predictors than measures indexing the sum of various syntactic operations (i.e., the number of opened, unified and kept open dependencies). This is an interesting finding as it suggests that unifying syntactic dependencies plays a pivotal role in predicting sentence comprehension difficulty (see also [19]). Since Uddén et al. did not report any analyses involving unif measures, it is unclear how well these would predict participants’ neural activity. Future research could address this question.

With regards to TP, our analyses showed that bigram TP (i.e., the probability of transitioning from one word to another in a forward or backward fashion) was a better predictor of self-paced reading times than trigram TP. The importance of bigram TP for reading has previously been highlighted in research using eye-tracking during reading ([65], see also [66]). Moreover, bigram TP has ties to the concept of ‘association strength’, either operationalized through free association tasks [67] or latent semantic analysis [68]. Associations are assumed to play an important role both in language comprehension (e.g., [69,70]) and cognitive processing [71] more broadly. The present analyses corroborate the role of bigram TP in language comprehension and showed that two-word contexts provided a better fit to reading times than three-word contexts. One may have predicted that a longer context may contain more information than a shorter context and that trigrams therefore should influence reading times more consistently than bigrams. Among others, effects of trigram TP have previously been reported in self-paced story reading [72]. One possibility is that bigrams were more important than trigrams in the present experiment because our participants read disconnected, isolated sentences rather than a semantically coherent story. Future research could explore under which conditions readers place more weight on bigrams and trigrams, respectively.

In our final analysis, we assessed the contributions of LB_unif, RB_unif, and forward and backward bigram TP to reading times when included in the same model. In doing so, we addressed the question whether SC measures explain variance in reading behavior over and above the TP measures (cf. [33,43]). While TP showed significant effects in all four models we conducted, significant effects of SC only emerged when the control variables were excluded.

Before discussing the implications of SC and TP effects in more detail, it is therefore important to highlight the role of the control variables. As it turned out, the control variables had consistent effects in all analyses and explained the bulk of variance in reading times: Participants took more time to read longer sentences (composed of more words) and longer words (composed of more letters) compared to shorter sentences and words. Word frequency had a negative effect with higher frequency resulting in shorter word reading times (see [73] for discussion of the effects of frequency in word processing). The strong length and frequency effects demonstrate that much of the variance in word reading times is associated with low-level word characteristics (rather than higher-level syntactic dependencies and lexical co-occurrence frequencies, cf. [51–53]).

At the word-level, we had additionally included position within the sentence as a control predictor (see Mak & Willems [50] for a similar approach). We observed that words later in the sentence were read more slowly than words at the beginning of the sentence. One account for this finding is that participants briefly scanned words at the beginning of a sentence, pressed the button quickly to bring up the next word, and took more time later in the sentence as they read the words *and* integrated the preceding lexical material into a sentence-model. Some support for this notion comes from reading research using electroencephalography. Van Petten and Kutas [74] found that words in a sentence, presented in rapid serial visual presentation at a fixed rate of 900 ms (200 on, 700 ms off), elicited smaller N400 components when occurring later as compared to earlier in the sentence. The authors took the inverse relationship between N400 amplitude and word position to reflect the growing influence of sentential constraints on word processing as a sentence builds up. The finding from the present analyses that word position consistently contributed to explaining variance in reading times highlights the need for including this measure as a control variable. In a way, these analyses also lend support for operationalizing the sentence-level RTs as a sum of word reading times, as such a measure may capture the cumulative effects of sentential constraints better than a dependent variable based, for example, on a minimum or maximum RT.

On a technical note, our analyses revealed important limitations when estimating the contributions of correlated predictors to a dependent variable. As became clear across the various analyses, the effects of our measures of interest changed drastically (in terms of size and directionality) when the control variables were included in the same model leading to multicollinearity (see e.g. [75] for a similar observation). To address the main goal of the present study (pitting SC and TP against each other), we ran models that only contained the measures of interest. Our final sentence- and word-level models, containing LB_unif, RB_unif, forward and backward bigram TP, revealed that all of the four variables contributed positively to reading times (at the sentence-level, the effect of bigram FTP failed to reach statistical significance). Taken together, the data thus provide some evidence for the claim that SC and TP jointly influence self-paced reading. However, when both sets of measures are included in models together with the control variables, the contributions of SC and TP appear to be trumped by the control variables.

Although the effects of SC and TP were smaller than those of the control variables, they must not be overlooked. The central question of this study was whether SC would explain variance over and above that of TP. The answer to this question appears to be ‘yes’ (when considered independent of the control variables). The picture that emerges is one where readers are sensitive to a multitude of cues, including low-level control variables (e.g., word length/frequency), more ‘global’ hierarchical information (i.e., syntactic dependencies distributed across the sentence) and local transitions between adjacent words during sentence comprehension. Such a multiple-cue account of sentence reading resonates well with proposals for other aspects of sentence comprehension (e.g., prediction [76,77]), where various cues contribute to comprehension and where the context in which language processing takes place determines how much weight is placed on which cue.

## Conclusion

The current study demonstrated independent effects of SC and TP on self-paced reading times, both at the sentence-level and at the word-level. With regards to SC, we observed that measures reflecting the number of a word’s syntactic unifications were better predictors than measures reflecting a multitude of syntactic operations (opening, closing and tracking an open dependency). In terms of TP, we showed an advantage of bigram over trigram measures in predicting variance in self-paced reading times. Throughout all analyses, we found strong effects of the control variables (e.g. sentence/word length, word frequency and word position), which explained the bulk of variance in our models. When comparing the small effects of SC and TP in the presence of the control variables, the effects of TP appear to be more robust.

## Supporting information

S1 File (DOCX)
