# Supplementary material for: Comparing predictors of sentence self-paced reading times: Syntactic complexity versus transitional probability metrics
Source: PLoS One. 2021 Jul 12;16(7):e0254546. doi: 10.1371/journal.pone.0254546 (PMC8274840; doi:10.1371/journal.pone.0254546)
Supplement: S1 File — (DOCX) [file pone.0254546.s001.docx]

**Appendix A: Syntactic complexity operationalization**

The dependency trees that were used to calculate the four syntactic complexity (SC) measures (LB, RB, LB_unif and RB_unif) are depicted in Figures 1 and 2 (for two example sentences). The trees were generated using an automated parser (FROG parser; Van den Bosch et al., 2007).


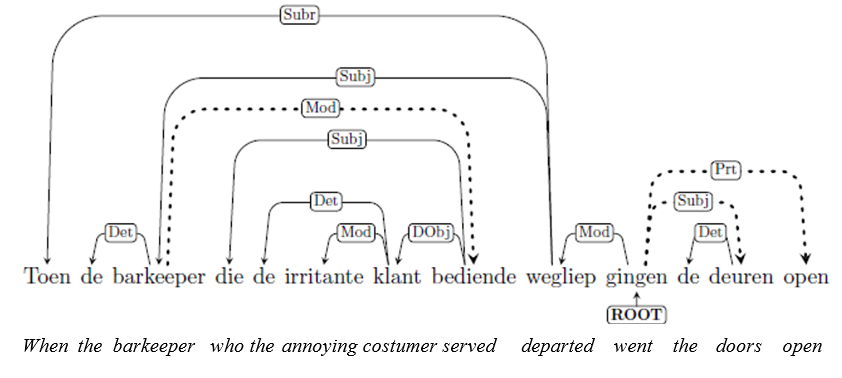


**Figure 1**. The dependency tree for the first example sentence (translation: When the barkeeper who served the annoying customer departed, the doors went open). Solid lines depict left-branching dependencies; dotted lines represent right-branching dependencies. Det = determiner; Mod = modifier; Subj = subject; Subr = subordinator; DObj = direct object; Prt = particle (or separable prefix of separable verb).


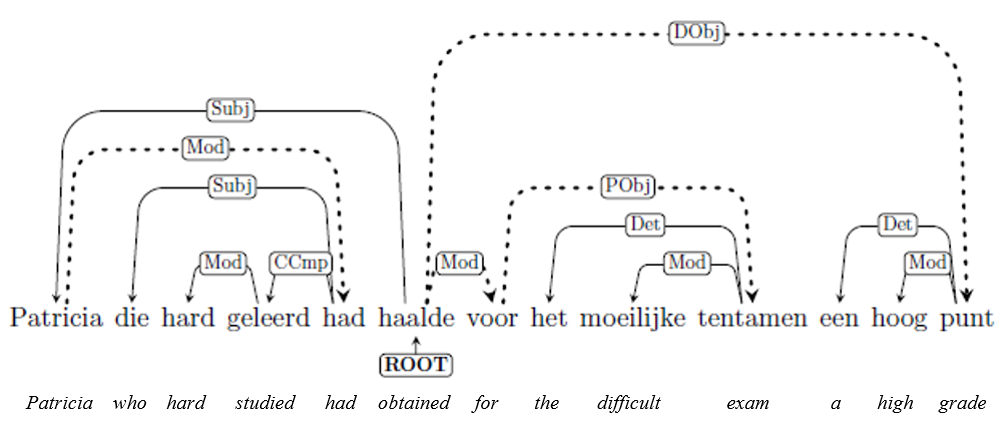


**Figure 2.** The dependency tree for the second example sentence (translation: Patricia who had studied hard obtained a high grade for the difficult exam). Solid lines depict left-branching dependencies; dotted lines represent right-branching dependencies. Det = determiner; Mod = modifier; Subj = subject; DObj = direct object; PObj = object of preposition; CCmp = clausal complement of verb.

The calculation of our four SC measures is graphically explained in the two figures below.

|  | Toen | de | barkeeper | die | de | irritante | klant | bediende | wegliep | gingen | | de | deuren | open |
| --- | --- | --- | --- | --- | --- | --- | --- | --- | --- | --- | --- | --- | --- | --- |
|  | *When* | *the* | *barkeeper* | *who* | *the* | *annoying* | *customer* | *served* | *departed* | | *went* | *the* | *doors* | *open* |
| Toen |  |  |  |  |  |  |  |  |  |  | |  |  |  |
| de |  |  |  |  |  |  |  |  |  |  | |  |  |  |
| barkeeper |  |  |  |  |  |  |  |  |  |  | |  |  |  |
| die |  |  |  |  |  |  |  |  |  |  | |  |  |  |
| de |  |  |  |  |  |  |  |  |  |  | |  |  |  |
| irritante |  |  |  |  |  |  |  |  |  |  | |  |  |  |
| klant |  |  |  |  |  |  |  |  |  |  | |  |  |  |
| bediende |  |  |  |  |  |  |  |  |  |  | |  |  |  |
| wegliep |  |  |  |  |  |  |  |  |  |  | |  |  |  |
| gingen |  |  |  |  |  |  |  |  |  |  | |  |  |  |
| de |  |  |  |  |  |  |  |  |  |  | |  |  |  |
| deuren |  |  |  |  |  |  |  |  |  |  | |  |  |  |
| open |  |  |  |  |  |  |  |  |  |  | |  |  |  |
| LB: | 1 | 2 | 3 | 3 | 4 | 5 | 6 | 4 | 3 | 1 | | 1 | 1 | 0 |
| RB: | 0 | 0 | 1 | 1 | 1 | 1 | 1 | 1 | 0 | 2 | | 2 | 2 | 1 |
| LB_unif | 0 | 0 | 1 | 0 | 0 | 0 | 2 | 2 | 2 | 1 | | 0 | 1 | 0 |
| RB_unif | 0 | 0 | 0 | 0 | 0 | 0 | 0 | 1 | 0 | 0 | | 0 | 1 | 1 |

**Figure 3**. Calculation of the four complexity measures for the first example sentence. Yellow rows depict left-branching depen-dencies; blue rows depict right-branching dependencies. In this sentence, the highest LB value occurs at the noun ‘klant’ (customer), because six left-branching dependencies are active at the occurence of that word. The verb ‘bediende’ (served) is the head of two left-branching dependencies: one with ‘die’ (who) as dependent, and one with ‘klant’ (customer) as dependent. Hence, two left-branching unifications occur at ‘bediende’, so this verb is assigned an LB_unif value of 2. Moreover, at the words ‘gingen’, ‘de’ and ‘deuren’, two right-branching dependencies are active. These are right-branching dependencies, because their head (‘gingen’ (went)) is encountered before the dependents. As a result, these three words are each assigned an RB value of 2. Lastly, at the word ‘deuren’ (doors), a right-branching dependency (with the verbal head ‘gingen’ (went)) is closed, meaning that ‘deuren’ is assigned an RB_unif value of 1.

|  | Patricia | die | hard | geleerd | had | haalde | voor | het | moeilijke | tentamen | | een | hoog | punt |
| --- | --- | --- | --- | --- | --- | --- | --- | --- | --- | --- | --- | --- | --- | --- |
|  | *Patricia* | *who* | *hard* | *studied* | *had* | *obtained* | *for* | *the* | *difficult* | | *exam* | *a* | *high* | *grade* |
| Patricia |  |  |  |  |  |  |  |  |  |  | |  |  |  |
| die |  |  |  |  |  |  |  |  |  |  | |  |  |  |
| hard |  |  |  |  |  |  |  |  |  |  | |  |  |  |
| geleerd |  |  |  |  |  |  |  |  |  |  | |  |  |  |
| had |  |  |  |  |  |  |  |  |  |  | |  |  |  |
| haalde |  |  |  |  |  |  |  |  |  |  | |  |  |  |
| voor |  |  |  |  |  |  |  |  |  |  | |  |  |  |
| het |  |  |  |  |  |  |  |  |  |  | |  |  |  |
| moeilijke |  |  |  |  |  |  |  |  |  |  | |  |  |  |
| tentamen |  |  |  |  |  |  |  |  |  |  | |  |  |  |
| een |  |  |  |  |  |  |  |  |  |  | |  |  |  |
| hoog |  |  |  |  |  |  |  |  |  |  | |  |  |  |
| punt |  |  |  |  |  |  |  |  |  |  | |  |  |  |
| LB: | 1 | 2 | 3 | 4 | 3 | 1 | 0 | 1 | 2 | 2 | | 1 | 2 | 2 |
| RB: | 1 | 1 | 1 | 1 | 1 | 2 | 3 | 2 | 2 | 2 | | 1 | 1 | 1 |
| LB_unif | 0 | 0 | 0 | 1 | 2 | 1 | 0 | 0 | 0 | 2 | | 0 | 0 | 2 |
| RB_unif | 0 | 0 | 0 | 0 | 1 | 0 | 1 | 0 | 0 | 1 | | 0 | 0 | 1 |

**Figure 4**. Calculation of the four complexity measures for the second example sentence. Yellow rows depict left-branching depen-dencies; blue rows depict right-branching dependencies. In this sentence, at the verb ‘geleerd’ (studied), four left-branching dependencies are active, so the LB value of this verb is 4. This verb is also the head of one left-branching dependency, with ‘hard’ (hard) as dependent. Thus, one left-branching unification occurs at ‘geleerd’, meaning that it is assigned an LB_unif value of 1. Moreover, at the word ‘voor’ (for), three right-branching dependencies are active, meaning that this word is assigned an RB value of 3. Since it is also the dependent of a right-branching dependency with ‘haalde’ (obtained) as its head, ‘voor’ is given an RB_unif value of 1.

**Appendix B: Linear mixed-effects model formulas and model diagnostics**

Description of all mixed-effects model formulas, accompanied with each model’s diagnostics, obtained using the ‘performance’ package and ‘check_model’ command in R (Lüdecke et al., 2020). The model diagnostics include visual checks of various model properties and assumptions, including normality of residuals, normality of random effects, heteroscedasticity, homogeneity of variance and multicollinearity.

Control models:

control_model_sentence = lmer(RT_sum_log ~ 1 + NWords_cs + sum_Zipf_cs + (1|Subject) + (1|sentence_idx), data = data, control=lmerControl(optimizer="bobyqa"))

control_model_word = lmer(word_RT_log ~ 1 + word_length_orth_cs + Zipf_cs + word_position_cs + (1|word_idx) + (1|Subject), data = data_word, control=lmerControl(optimizer="bobyqa"))

SC models: sentence-level:

*model_SC_1 = lmer(RT_sum_log ~ 1 + NWords_cs + sum_Zipf_cs + sum_LB_cs + sum_RB_cs + (1|Subject) + (1|sentence_idx), data = data, control=lmerControl(optimizer="bobyqa"))*

*
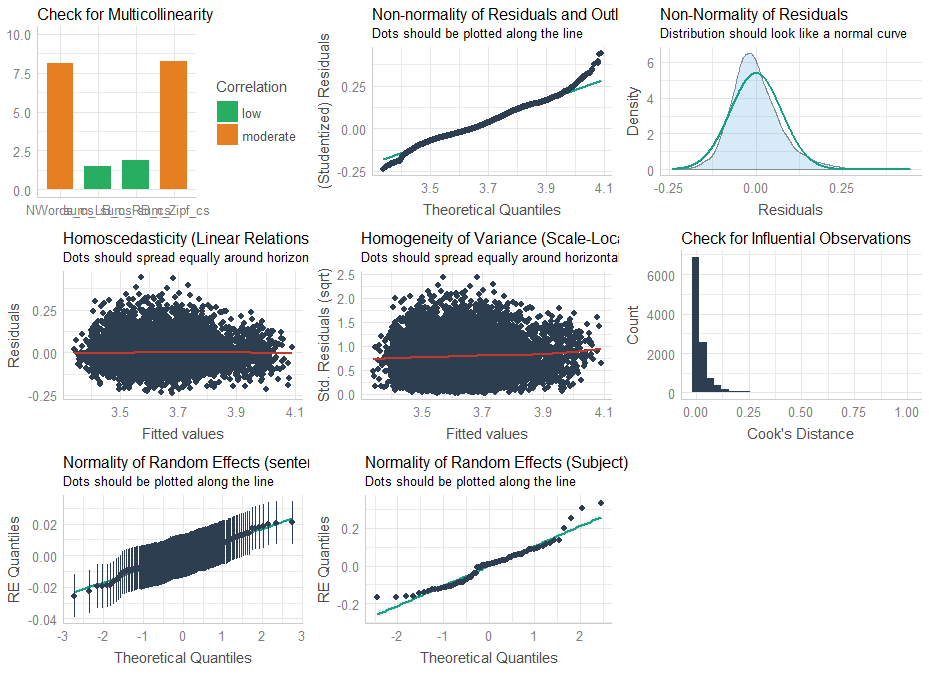
*

*model_SC_2 = lmer(RT_sum_log ~ 1 + NWords_cs + sum_Zipf_cs + sum_LB_unif_cs + sum_RB_unif_cs + (1|Subject) + (1|sentence_idx), data = data, control=lmerControl (optimizer="bobyqa"))*

*
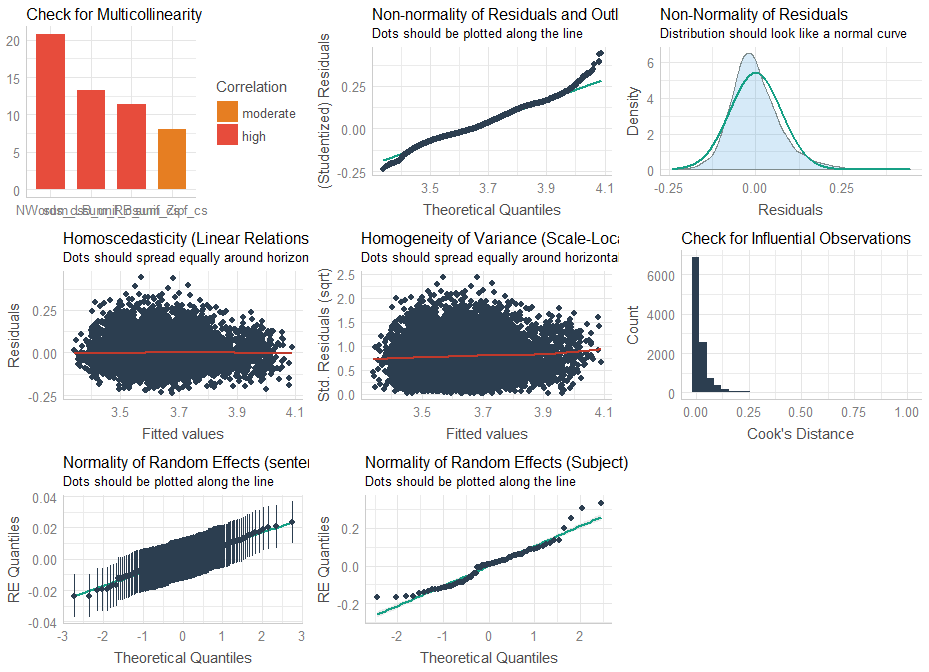
*

SC models: word-level:

*model_word_SC_1 = lmer(word_RT_log ~ 1 + word_length_orth_cs + Zipf_cs + word_position_cs + LB_cs + RB_cs + (1|word_idx) + (1|Subject), data = data_word, control=lmerControl (optimizer="bobyqa"))*

*
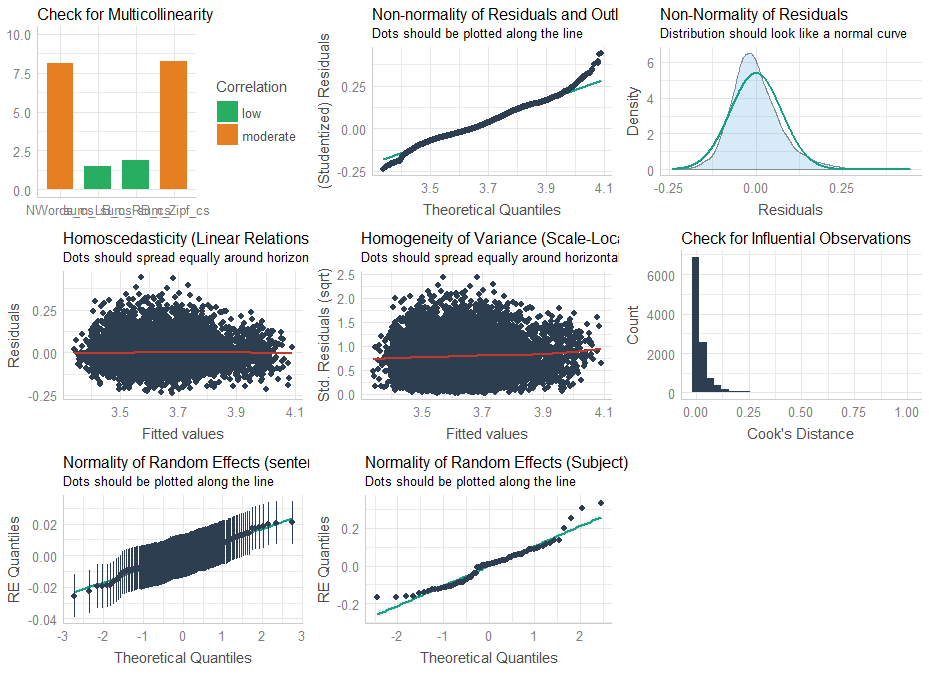
*

*model_word_SC_2 = lmer(word_RT_log ~ 1 + word_length_orth_cs + Zipf_cs + word_position_cs + LB_unif_cs + RB_unif_cs + (1|word_idx) + (1|Subject), data = data_word, control=lmerControl (optimizer="bobyqa"))*

*
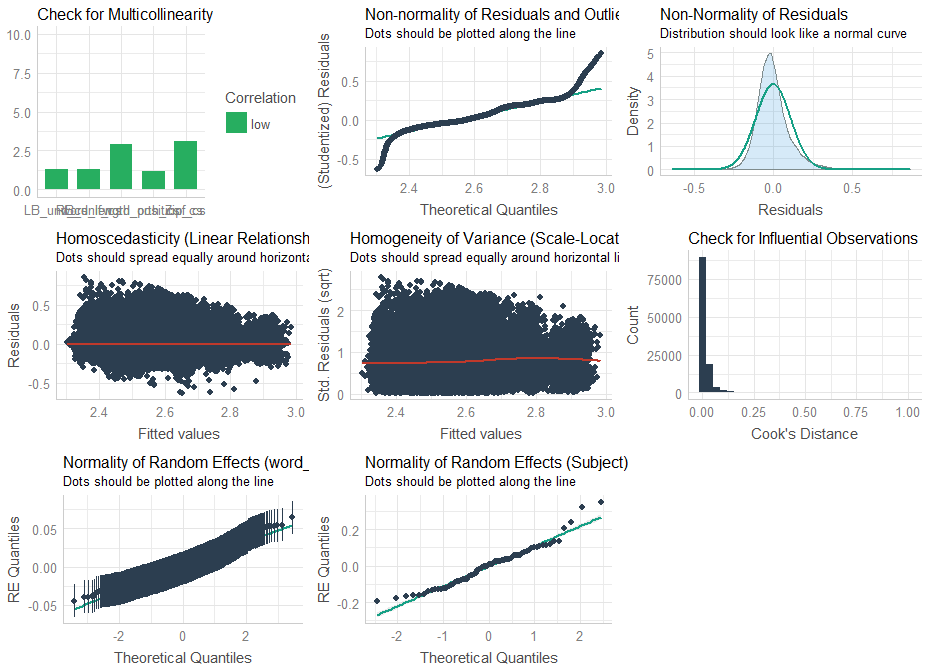
*

TP models: sentence-level:

*model_TP_1 = lmer(RT_sum_log ~ 1 + NWords_cs + sum_Zipf_cs + sum_ftp_bigram_cs + sum_btp_bigram_cs + (1|Subject) + (1|sentence_idx), data = data, control=lmerControl (optimizer="bobyqa"))*

*
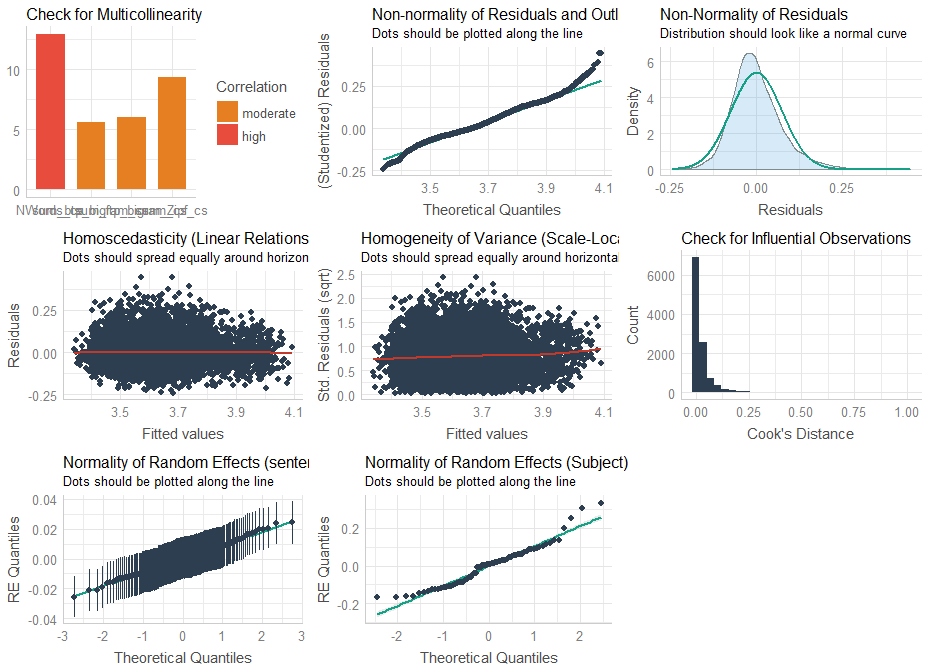
*

*model_TP_2 = lmer(RT_sum_log ~ 1 + NWords_cs + sum_Zipf_cs + sum_ftp_trigram_cs + sum_btp_trigram_cs + (1|Subject) + (1|sentence_idx), data = data, control=lmerControl (optimizer="bobyqa"))*

*
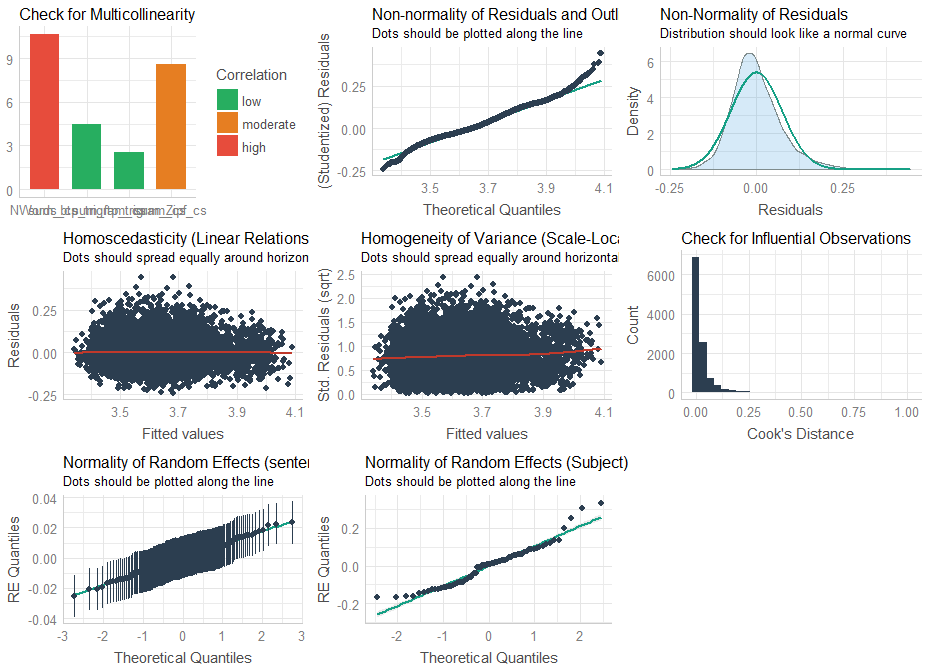
*

TP models: word-level:

*model_word_TP_1 = lmer(word_RT_log ~ 1 + word_length_orth_cs + Zipf_cs + word_position_cs + ftp_bigram_word_cs + btp_bigram_word_cs + (1|word_idx) + (1|Subject), data = data_word, control=lmerControl(optimizer="bobyqa"))*

*
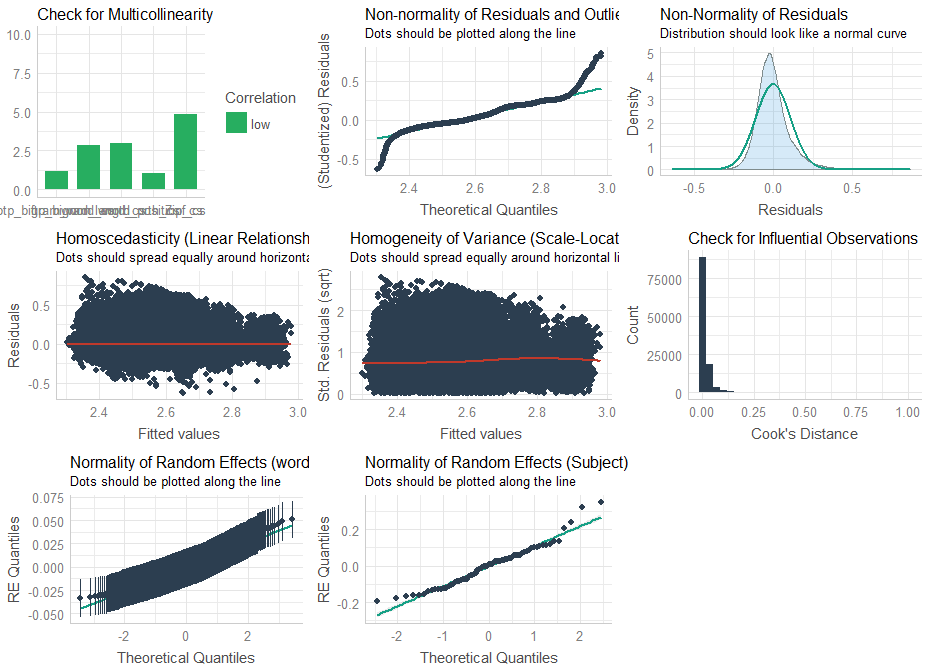
*

*model_word_TP_2 = lmer(word_RT_log ~ 1 + word_length_orth_cs + Zipf_cs + word_position_cs + ftp_trigram_word_cs + btp_trigram_word_cs + (1|word_idx) + (1|Subject), data = data_word, control=lmerControl(optimizer="bobyqa"))*


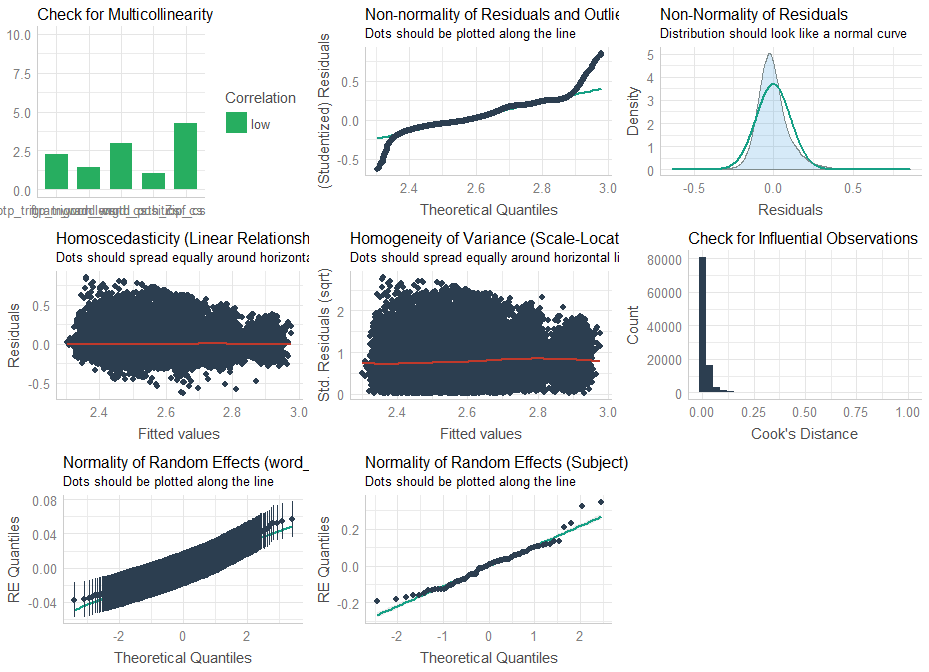


‘Full’ model: sentence-level:

*model_full = lmer(RT_sum_log ~ 1 + NWords_cs + sum_Zipf_cs + sum_LB_unif_cs + sum_RB_unif_cs + sum_ftp_bigram_cs + sum_btp_bigram_cs + (1|Subject) + (1|sentence_idx), data = data, control=lmerControl(optimizer="bobyqa"))*

*
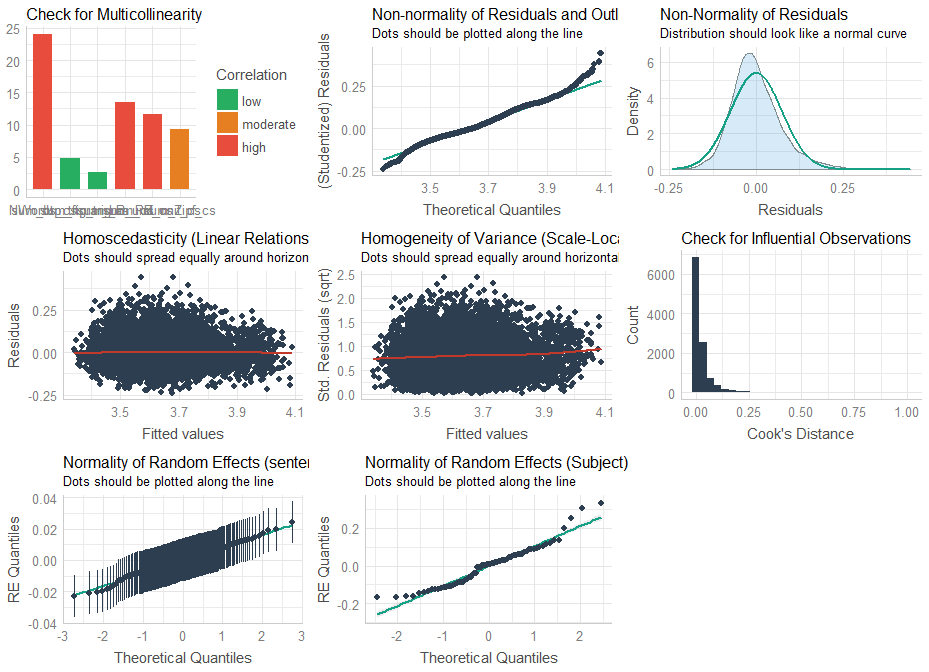
*

‘Full’ model: word-level:

*model_word_full = lmer(word_RT_log ~ 1 + word_length_orth_cs + Zipf_cs + word_position_cs + LB_unif_cs + RB_unif_cs + ftp_bigram_word_cs + btp_bigram_word_cs + (1|word_idx) + (1|Subject), data = data_word, control=lmerControl(optimizer="bobyqa"))*

**
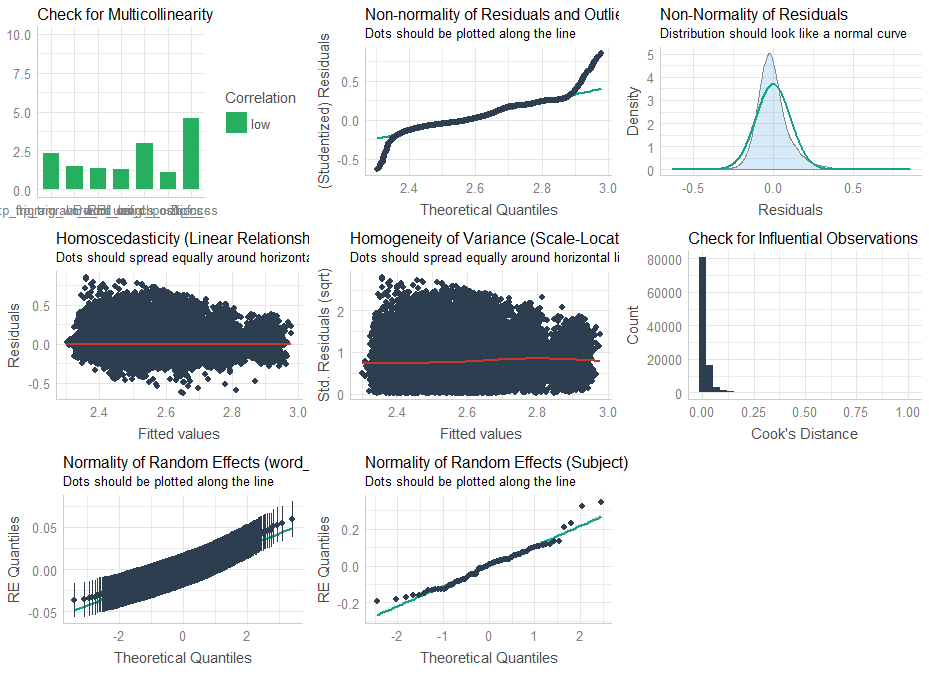
**
